# Alcoholic Liquors in the Practice of Medicine

**Published:** 1887-09

**Authors:** Eugene Foster

**Affiliations:** Augusta, Georgia


THE ATLANTA MEDICAL AND SURGICAL JOURNAL.

VOL. IV. SEPTEMBER, 1887. No. 7.

### Original Communications.

### ALCOHOLIC LIQUORS IN THE PRACTICE OF MEDICINE.

READ BEFORE THE MEDICAL ASSOCIATION OF GEORGIA AT ITS THIRTY-EIGHTH ANNUAL SESSION IN ATLANTA, APRIL 21, 1887, BY EUGENE FOSTER, M. D., AUGUSTA, GEORGIA.

(CONTINUED FROM PAGE 337.)

The paper under review says: "It can hardly now be doubted that alcohol is the real principle upon which medical reliance is placed in all these compounds which go under the general term of spirituous liquors."

If the above paragraph be intended to maintain that alcohol is the most important of any one ingredient in the various alcoholic liquors, then I fully endorse the statement. But if it be intended to even intimate that alcohol possesses all the virtues of alcoholic liquors, then I unhesitatingly and positively deny the statement.

To the contrary, I assert that the following remarks from "Stillé's Therapeutics and Materia Medica" is a correct, uncontroverted and incontrovertible statement of the status of medical opinion upon this question as enunciated by the best, most authoritative and most advanced therapeutists known to our profession: "The action of wine is essentially stimulant. This property it derives chiefly from its alcoholic element, but in a degree which is far from being measured by the proportion of the latter, and it is further so completely modified by the other elements contained in the liquid as to render the direct and still more ulterior effects of wine widely different from those of ardent spirits. This peculiarity appears to be derived from the salts, acids, sugar and ethereal elements in which it abounds. . . . . . Hence it is incorrect to suppose that the alcohol contained in any given specimen of wine represents precisely its intoxicating power, or to assert, as some have unwisely done, that because a wine contains a certain percentage of alcohol its effects upon the system will be identical with those which a like amount of alcohol diluted with water alone would produce."

This is a most important question to the members of the profession. Prohibitionists contend that when alcohol and domestic wines are allowed for prescription by physicians they have accorded us all that we can reasonably ask, and that they have, in so doing, been fully considerate of the sick, at the same time they have saved our people from the frightful evil of intemperance. A brief consideration will show the absurdity of the present local option law. Those of you who practice medicine in Atlanta may desire and feel compelled to prescribe whisky, brandy, gin, rum, beer, or the finer grades of imported wines, to patients under your professional care, and yet you cannot do so without making criminals of yourselves, for your law says that only domestic wines for the sick and alcohol for medicinal, art or mechanical and scientific purposes can be sold. Now take domestic wines. Dr. Logan quotes and endorses the following language of Dr. B. W Richardson: "To prescribe wine without knowing whether the specimen of wine contains ten, fifteen or twenty per cent. of alcohol, to prescribe spirits without knowing the per cent. of alcohol, or whether all the alcohol in the spirits is ethylic alcohol or a mixture of alcohols; to prescribe either wines, spirits or ales without asking whether other chemical bodies than alcohol are or are not present in them is not prescribing at all." Now, sir, let me ask if you know what percentage either of alcohol, volatile oils, œnanthic ether, tannic acid, malic acid or other acids, bitartrate of potassa, etc., which is contained in any wine made in Georgia. You are compelled, sir, to answer, I do not, and the same reply must be made by every intelligent, honest physician in this State. If this be so, and it cannot and will not be denied, then when these local option laws permit you to use domestic wines in sickness, you, under the statement of Dr. Logan, cannot prescribe them for your patients because you do not know what percentage of alcohol and other substances they contain. Not one physician in a thousand has the time or ability to make chemical analyses of the wines used in our practice; nor is there a vineyard in Georgia where the vintage is extensive enough to bear the expense of chemical analysis by a learned chemist. Do we not know that the percentage of ethylic alcohol in different wines varies with the age of the wine, as well as the locality and season in which the grapes have grown, and also on the one hand on the quantity of sugar in the grape, on the other upon the degree of fermentation in the "must?" Notwithstanding this fact, we are expected to rest content with prescribing wine made from a few mashed blackberries or grapes made into a vintage by the backwoods crackers of this Empire State of the South. If you wish and prefer to prescribe the better wines from the Rhine—Main and Moselle, or Malaga, Madeira or Port, or Champagne, etc.—you cannot do it, for your considerate and wise local option law says, "Stop, you cannot prescribe these wines, for they are imported goods." The intention of this law, enacted by the learned and highly scientific members of the Georgia Legislature, was probably to protect the enormous wine-growing industry at present existing in this State, where a few rustics, wholly ignorant of the elemental principles of chemistry, make a two-gallon jug of sour wine, and complacently congratulate themselves and the commonwealth upon the magnitude of their enterprise. Do you not know that the better imported wines have been repeatedly submitted to chemical analysis, and the proportions of ethylic alcohol, ether, acids, etc., ascertained with such definiteness as to enable us to know the probable proportions or percentage of these substances in any one of these wines as their names are called ? If you doubt it, turn to your text-books and see if this statement is not true. So we see that those of us who contend for the liberty of intelligent exercise of judgment in prescribing wines founded upon chemical demonstration of their constituents, come they from whence they may, are endeavoring to do the very thing which Drs. Richardson and Logan say is essential to intelligent, rational prescription of the remedy; yet if we follow the local option law, we are compelled to violate every element of the demands of these honorable and learned men.

Well, probably the answer is ready by saying, "Prescribe ethylic alcohol, and these uncertainties vanish, and your patient is benefited; at the same time drunkenness is decreased to the greatest possible extent." Such an answer would be absurd. If the local option bill of this State were properly named it would be headed about as follows : "An Act intended to slander the medical profession, oppress the sick, and make drunkenness easy, more economical and destructive in Georgia." Such a title to this bill would be proper for the following reasons: 1st. The want of confidence in the medical profession is unfounded and slanderous. 2nd. By general consensus of medical testimony, I have shown that alcohol and domestic wines are not full, reliable substitutes for all alcoholic liquors; this being so, the sick are oppressed by denying them the best remedy in our *materia medica.* 3rd. This bill facilitates drunkenness in Georgia for the following reason: While the whole medical profession, speaking by the text-books, deny that alcohol is a full substitute for all alcoholic liquors in disease, nobody denies that alcohol is the intoxicant ingredient of all alcoholic liquors. I favor putting alcoholics in the prescription case of reputable licensed druggists for dispensing, under wise restrictions, upon the prescription of practicing physicians, as is done with other deadly poisons, opium, morphine, arsenic, strychnia, prussic acid, etc. The Solomons of the Georgia Legislature have, in effect, said: never, for the doctors and druggists may transfer the bar-rooms—which we seek to destroy—to the druggists' counters. Indeed, some over-zealous prohibitionists have openly made the charge that our profession will do so if the opportunity be presented. Such an assault upon the integrity of the medical profession is wholly gratuitous—absolutely false, and could only emanate from a fearfully depraved mind. Go into every community in the world and select the men who are noted for high sense of honor, humanity, self-sacrifice and love of law and order, and you will find that the physicians of every community are held in highest esteem, and are more trusted than any other body of men therein. I am willing to admit that individual members—few in number—may prove recreant to their high trust, and connive with druggists in furnishing alcoholics to those not needing them for medicinal purposes, but such doctors would be regarded by the medical profession in the same light as the Legislature would look upon one of their number who would sell his honor and his services to the villain who attempts to lobby a dishonest bill through the Legislature. No, sir, the medical profession, whose watchword is duty, whose calling demands the exercise of the very highest type of unselfishness—who never turn a deaf ear to the cry of suffering humanity, whether from prince or pauper—whose greatest joy is to succor the fallen and helpless and bring them back to manhood's estate—whose members heroically face death in a thousand forms to cure the pains of disease afflicting their fellow-man—whose members, by their charity, heroism, unselfish devotion to duty, have illustrated all the highest and best attributes of manhood in the sight of God and man, should not be so gratuitously slandered.

But, for the sake of the argument, let us do our noble profession the injustice to suppose that we are ready to join with druggists in an effort to transfer the bar-rooms to the drug-stores.

Look, for a moment, at the frightful mental imbecility evidenced in the local option bill—if that bill was intended as a remedy for this evil. The local option bill does not deny doctors the right to prescribe alcohol for medicinal purposes. If the doctors wish to act as agents to gratify the appetites of the people for strong drink, can they not prescribe alcohol made palatable to the toper? We are unworthy to be trusted to prescribe whisky, brandy, gin, rum, imported wines, etc., but by this law we are allowed to prescribe alcohol, and can, if we are mean enough to do it, make every man, woman and child in the commonwealth drunk by having them use a palatable mixture of ethylic alcohol. But this is not the only weak point in the "local option bill." The druggist, under this law, can, without the physician's prescription, sell alcohol to every man in the State who calls for it and says he wishes it for scientific, art or mechanical purposes. How does the druggist know what the buyer intends to do with alcohol after he purchases it? The mere statement of the buyer is proof sufficient under the law that he wishes it for scientific, art or mechanical purposes. The only art which may be involved in the trade is the dexterity of the purchaser in devising and effecting his purpose to get strong drink by telling a disgraceful lie; the only mechanical use which he may desire to make of the alcohol is to feed a vitiated appetite for strong drink, and alcohol is his panacea. I have been asked, "Will a man drink alcohol if he cannot get alcoholic liquors?" Certainly he will. I have known a man, whose wonderfully brilliant mind was the pride of his friends, and whose integrity was the boast of his community, fail to induce dealers to sell him liquor contrary to the request of his friends, and I have seen him steal and drink alcohol kept in the village physician's office for making tinctures. The local option bill makes drunkenness more deadly by forcing the inebriant to drink strong alcohol because he cannot get it in the diluted and less injurious form of alcoholic liquors. Do we not know that the drinking of alcoholic liquors is more injurious in proportion to the increase of the amount of alcohol in the compound? This point is certainly undeniable.

The essay under review says:

"Thus we see the great upheaval which is beginning in the medical profession, the result of which, in my judgment, will produce an entire revolution in the present practice of physicians in the use of alcohol. What part shall we bear in this great professional reform, upon which so much that is vital to the race depends? Shall we throw obstacles in the way? Shall we stand idly by, or, pursuing the same old beaten track, refuse to recognize the facts which are becoming so plain that he 'who runneth may read,' or shall we come up to the full measure of our opportunities and obligations as physicians, as conservators of the public health, as philanthropists, as citizens, as men who acknowledge our accountability to conscience and to God?"

Gentlemen, what is the extent of this mighty upheaval of professional opinion, which the essay under review shows us is bursting upon the medical profession in relation to the use of alcoholic liquors in the practice of medicine? Here it is: The individual views of Dr. N. S. Davis, of Chicago, Illinois, who sometimes prescribes alcoholic liquors in disease, and who has for a quarter of a century in vain labored to induce the medical profession to adopt his opinion; the views of Dr. James R. Nichols, of Haverhill, Massachusetts, who, in the same paper, in commenting on the statement of Dr. Holmes, "that if the contents of our drug-stores were taken out upon the ocean and thrown overboard, it would be better for the human race, but worse for the fishes," says, "this statement may be a little too sweeping;" the declaration of two hundred doctors in Brooklyn, New York, truthfully, manfully, and properly saying, "We would welcome any judicious and effective legislation, state or national, which should seek to confine the traffic in alcohol to the legitimate purposes of medical and other sciences, art and mechanism." The united voices of two thousand physicians of Great Britain, recognizing their duty as "conservators of the public health," saying, "that total and universal abstinence from all alcoholic beverages (mark you, not from medicinal use) of all sorts would greatly contribute to the health, prosperity and happiness of the human race."

This, gentlemen, so far as it has been pointed out, is the sum total of "the great upheaval of professional opinion which will produce an entire revolution in the present practice of physicians in the use of alcoholic liquors in disease." What question in science has ever been revolutionized upon any such testimony as this ? Is it upon such a narrow circle of individual speculations and negative opinions, contravening the positive experience and rich results of practically the entire medical profession of all countries, as testified to by an almost unbroken line of text-writers for the past quarter of a century, that we are to be forced to expunge from our materia medica one of the most valuable therapeutic agents known to the healing art ? Is it upon such speculations as contained in the views of Drs. Davis and Nichols that we are to be called upon by temperance crusaders to array ourselves upon the side of the local option bill of this State—a law which is an outrageous assault upon the integrity and rights of the medical profession, a cruel, inhuman oppression of the sick and afflicted under our professional care—a bill which is admitted by the author of the essay to be imperfect and not free from objections—a law which is so unjust and repulsive to thinking people that, while we were promised that it would drive intemperance from our grand old State, is not and cannot be enforced in prohibition counties ? At the last session of the Legislature Dr. Felton, who is the Moses of the temperance crusade, and who was expected to lead our people out of the valley of drunkenness into the land flowing with water and milk, startled our credulous citizens by solemnly declaring that in prohibition counties the law was null and void, because the law allowed the sale of domestic wines. The following is an extract from Dr. Felton's speech at the last session of the Legislature, to which reference has been made :

"THE SPECIAL ORDER.

"Mr. Felton, of Bartow, moved to take up the bill levying a tax of $10,000 upon dealers in domestic wines.

"Mr. Russell, of Clarke, moved to lay the motion on the table, but withdrew it to allow Mr. Felton to address the House.

"Mr. Felton said the bill contained no prohibition. Its sole object was to enforce the law in prohibition counties. He contended that the prohibition law was null and void in most counties because of the fraudulent practices of anti-prohibitionists. He insisted that the bill should be taken up and disposed of.

"Mr. Felton spoke scathingly of the wine-rooms. In giving birth to local option the General Assembly had given birth to a new nomenclature—'the wine-rooms.' All he asked was that the law should be enforced. Under the exemption of domestic wines all sorts of devices had been resorted to for the purpose of cheating and evading the law. He had voted to exempt domestic wines because he understood wine to be the fermented juice of the grape. A man might drink a great deal of such wine without being driven to murder, to suicide, and to all sorts of dreadful crimes. He had been mistaken. He had awaked to the fact that wine now meant a few bushels of blackberries, a few barrels of apples, a few baskets of peaches, 1,000 hogsheads of water and the poisoned contents of two drug-stores poured over all. [Applause and laughter.] Men claiming immunity for their vested rights manufactured and sold this poisonous concoction.

"The temperance people appealed, in the name of humanity and of law, to be delivered from such an outrage. This was the object of the bill.

"Speaking of the word domestic, Mr. Felton said that he had thought he knew its meaning. He was mistaken. Under the definition of the wine-dealers and their lawyers it meant to-day, anywhere in the United States [laughter]; wines shipped from California were bottled and sold as domestic wines. Wines shipped from all over the world were similarly treated.

"The temperance people asked to be protected from violations of the law by the passage of the bill."

What a pitiable spectacle is here presented! The assembled wisdom of the State enacted the local option bill, and went home satisfied that in so doing they had arrived at the mountain from which they could complacently view the promised land of temperance. They go home and put the law in operation in every county in Georgia, except 17, I believe. Now for the results: At the next session of the Legislature—two years from the enactment of the law—Dr. Felton, the intellectual giant of Georgia, who never quailed before the unterrified organized democracy of Bartow and routed them, horse, foot and dragoon, whenever they attempted to relegate him to private life, and who wields justly, I am free to confess, a power of influence in Bartow which no other dozen men in the county possess, comes to the assembled wisdom of Georgia, and frankly admits that the "local option bill," long since adopted in Bartow county, is not and cannot be enforced, and said, "The temperance people appealed, in the name of humanity and of law, to be delivered from such an outrage." Why come to the Legislature asking the enforcement of this marvelously perfect edict? He knew, and you know, that the Legislature is a legislative, not an executive body. The courts of his county and judicial district, panoplied with all the authority of this State, were open, and able to suppress all violations of law in their jurisdiction, provided public sentiment in the county demanded the enforcement of the law. Why leave the courts of his circuit, the judge, who, with the high sheriff of the county and his deputies, with a learned solicitor, provided by the State, free of expense to the temperance people, to prosecute violators of the statute, anxious and ready to enforce it. And yet Dr. Felton leaves the prohibition law and appeals to the Legislature, "in the name of humanity and of law, to be delivered from such an outrage." Delivered how? Not by the vaunted "local option bill," but by the remedy of high license, $10,000 per annum. Dr. Felton seems to have just learned that a law which is not approved by the judgment of the great mass of the people cannot and will not be enforced. Why, gentlemen, the statute-books are full of laws which are never enforced, actually existing as so many dead letters, and why? Because the people, who in this government rule, are satisfied that in consequence of some serious defect or evil of the bill the cause of justice is best advanced in "honoring such law more in the breach than in the observance of it."

What part shall we, physicians of Georgia, bear in this great reform, upon which so much that is vital to the race depends? "Shall we throw obstacles in the way?" I unhesitatingly and emphatically answer, self-respect, professional dignity, consideration for the sick and afflicted, duty to the public in teaching the honestly ascertained facts bearing upon the medical aspects of the question, responsibility to conscience, to man and to God will not permit us to be neutral or silent. We must "come up to the full measure of our opportunities and obligations as physicians, as conservators of the public health, as philanthropists, as citizens, as men who acknowledge our accountability to conscience and to God," and, with all the emphasis possible, declare that we cannot, will not support any such law as this, for it is a gross usurpation of power to deny to the physician the right to prescribe whatever remedy for his patient that the intelligent judgment of the medical profession endorses as either advantageous or necessary in the cure of disease. We cannot, must not accept this "local option law." There is a great principle involved in this issue, and we should stand for it as immovably as the rocks and "everlasting hills"—that great principle which we should and must contend for is that to the medical profession belongs, necessarily, solely, absolutely, the right to use any medicinal agent which, in the opinion of the profession, is either serviceable or necessary to the preservation of the health or prolongation of the lives of those who commit themselves to our professional care. No matter how dangerous such a remedy may be if abused by the public when not used as a therapeutic agent, no clamor of the public, no claptrap oratory of the political demagogue, no Utopian views of the fanatic, should ever be allowed to deprive the medical profession of the use of it so long as it is prescribed solely as a therapeutic agent. Once grant to the public the right to say what the medical profession shall or shall not prescribe as a medicine for the relief of disease, and you thereby cravenly surrender to them your inherent right to the exercise of liberty of intelligent judgment and action, and thereby surrender to the political mountebank, or the fanatic, the right to think and act for you, who alone are qualified to intelligently and honestly decide medical questions. Once surrender your rights to prescribe any kind of alcoholic liquors as therapeutic agents because the lying drunkard—seeking an excuse by which to palliate his own shame and disgrace—falsely accuses our profession of having been the author of his fall by prescribing alcoholics for his diseased body, you thereby acknowledge your recreancy to a high and honorable trust, and invite the public to further invade the temple of medicine and take from us the right to prescribe chloroform, ether, hydrate of chloral, opium, morphine, laudanum, paregoric, and many of the most indispensable medicines known to our materia medica. Do you not, and the public also, know that thousands and hundreds of thousands of men and women of all classes of society all over this land are either frequently or habitually drunk on chloroform, ether, opium in its various forms, hydrate of chloral, cocaine, etc.? Are you willing to admit, because some or all of these men and women were suffering from disease, and members of our profession found it necessary to administer chloroform, ether, opium, hydrate of chloral, or cocaine for the relief of pain or to procure needful sleep, that this temporary use of these indispensable remedies makes us justly chargeable with being the cause of the evil, disgraceful habits into which these unfortunate beings have fallen?

Should we not, in justice to fairness and truth, deny that the prescription of these therapeutic agents was honestly blamable for the misfortune which has fallen upon these people? Truth, honesty, justice demands that, in attempting to find the cause of these misfortunes, we should say that as a rule these unfortunates have but perverted the beneficent gifts of science to their wanton purposes by abusing instead of properly using these God-given remedies. If charity must be brought to cover their weaknesses, be it so; but do not slander the medical profession, for it is innocent of the wrong; rather seek the cause of the evil in the probability that these substances supply these people with some natural need of their system, or some craving of their vitiated constitutions. Gentlemen, no matter how earnestly we would wish it were otherwise, the fact remains that there is undeniably an inborn craving of the human constitution for stimulants and narcotics. This statement is verified by the history of all peoples, both civilized and uncivilized, for we find that fully five hundred millions of the human race habitually drink infusions of tea; one hundred millions drink infusions of coffee; fifty millions use cocoa; ten millions use an infusion of mate or guarana; four hundred millions smoke or eat opium; three hundred millions haschisch or Indian hemp; a majority of all the peoples of the earth smoke or chew tobacco, or use alcoholic liquors as a beverage. When you interrogate philosophy for the reason of this well-nigh universal habit of using stimulants and narcotics by all peoples of the earth, the only answer which the profoundest philosopher can give is that "it looks like the influence of some deep-seated necessity of the human constitution which our philosophy is unable to fathom, and which science has yet to explore." It would probably be well, just here, for temperance crusaders to tell us why "Dame Nature" has been guilty of the caprice of making every man, woman and child on earth tee-totalers as well as drunkards—brew alcohol in his or her economy, and why alcohol—this deadly poison to human health, morals and prosperity—is the only beverage used by mankind which is naturally brewed in the human system. This fact, gentlemen, seems to point with unerring certainty to the additional fact that alcohol was intended by God Almighty to serve some profound physiological purpose, and supply some want of the human economy when wisely and properly used. It would be absurd to argue, because alcoholics are harmful when abused, that they are necessarily baneful to the human constitution when judiciously used.

"The physician," says Richardson, "is often called upon to witness symptoms of diseases which actually are produced by the improper use of the instruments of cure he discovers or invents for the good of the world. The commonest instruments thus misapplied in our life of to-day are opium, chloral-hydrate, chloroform, ether, the compounds sold under the name of chlorodyne and absinthe." The physician is fully aware of the tendency of the human body to resort to the habitual use of stimulants and narcotics, and he should always, under all circumstances, bear in mind and put in practice the following words of wisdom uttered by the two hundred leading practitioners of London, England: "The undersigned, while unable to abandon the use of alcohol in the treatment of certain cases of disease, are yet of opinion that no medical practitioner should prescribe it without a sense of grave responsibility. They believe that alcohol, in whatever form, should be prescribed with as much care as any powerful drug, and that the directions for its use should be so framed as not to be interpreted as a sanction for excess, or necessarily for continuance of its use when the occasion is past." I endorse every word of precaution in the above extract. To show the readiness of human beings to pervert the use of medicines, I call your attention to the fact that within six months after hydrate of chloral had been introduced into medical practice in England, Richardson had met with so many instances of the morbid habit of habitual use of this valuable remedy by non-professionals that he uttered a public warning to the people on this subject. Every word of warning contained in the declaration of the two hundred leading practitioners of medicine of London in relation to the medical use of alcoholic liquors applies with equal justice and force to the prescription of all other stimulants and narcotics.

Finally, gentlemen, I protest against the attempt of temperance fanatics to force the physicians of Georgia into support of the "local option law" by charging that those of us who cannot, will not support it, are the friends and advocates of the bar-room liquor traffic. Every such charge is unfair, unmanly, dishonorable, maliciously false. You, I, every physician known to me in Georgia, warmly sympathize with, and will join them in any effort to obtain reasonable legislation tending to decrease needless temptations to drink alcoholic liquors as a beverage in common use among our people. For myself, I regard the attempts of temperance crusaders to coerce us into the support of the "local option bill," or class us as advocates of bar-rooms, as an imbecile, disgraceful farce—a contemptible effort at terrorism. The duty of the hour, upon the part of the physicians of Georgia, is plain. We should demand of temperance advocates to give us a law founded upon wisdom, justice and moderation, and we will accept and support it. But so long as the "local option law"—so unjust to the medical profession, so inhumanly inconsiderate of the sick, who place their health and lives in our professional charge—is continued in its present policy, we should meet the emergency like men; we should fight it at every opportunity, and demand its modification or rejection by the people; we should indignantly, disdainfully defy every effort at terrorism.

(*Concluded.*)

